# Pre- and Post-Interventional Changes in Physiological Profiles in a Patient Presenting With Opioid Withdrawal After Intrathecal Drug Delivery System Failure Related to Assumed Catheter Microfracture

**DOI:** 10.7759/cureus.14835

**Published:** 2021-05-04

**Authors:** Reza Ehsanian, Eugene Koshkin, Aleyah E Goins, Marena A Montera, Sascha Alles

**Affiliations:** 1 Orthopaedics and Rehabilitation, University of New Mexico School of Medicine, Albuquerque, USA; 2 Anesthesiology and Critical Care Medicine, University of New Mexico School of Medicine, Albuquerque, USA

**Keywords:** pain management, chronic pain, pain, narcotics, analgesics, microfracture, intrathecal drug delivery systems, opioid withdrawal, induced pluripotent stem cells, electrophysiology

## Abstract

The intrathecal drug delivery system (IDDS) is successfully utilized for the treatment of chronic pain conditions; however, they are associated with complications related to human error and system failure. A case report is presented of a patient with opioid withdrawal (OW) secondary to assumed catheter microfracture. Interrogation of the IDDS allowed for the collection of pre- and post-treatment/stabilization cerebrospinal fluid (CSF), which is used to investigate the possible physiological determinants of OW.

A 46-year-old female with a history of low back pain after traumatic low back injury status post-IDDS placement for failed back surgery syndrome presented with signs and symptoms concerning for OW. After every other possible explanation was ruled out, it was hypothesized that there may be IDDS catheter microfracture(s), and catheter replacement led to symptom resolution. There were no significant differences in cytokine levels tested in pre-CSF versus post-CSF samples. Whole-cell patch-clamp electrophysiology analysis of human-induced pluripotent stem cell-derived nociceptors after treatment with pre- and post-CSF samples demonstrated modulation of action potential waveform.

In patients presenting with acute OW attribution IDDS malfunction, catheter microfracture must be in the differential, and non-conventional interrogation of the IDDS catheter should be considered. The possible differences in pre-CSF and post-CSF may be more complicated than previously postulated, as there were no significant differences in cytokine profiles; however, treatment of in vitro neurons with pre- and post-CSF resulted in differential neuronal excitability, which may account for some of the symptoms of OW.

## Introduction

Chronic pain conditions, including lower back pain (LBP), considerably impact the lives of patients and account for significant health care expenditure [1]. LBP in particular is documented to be the leading cause for years lived with disability in the world [2]. The intrathecal drug delivery system (IDDS) is a safe and cost-effective therapeutic choice to treat those patients with chronic pain who have failed to show improvement with less invasive interventions [3-5]. Although relatively rare, IDDS complications occur and are attributed to intrathecal pump (ITP) and catheter malfunctions [6,7]. Catheter malfunctions include granuloma formation at the tip, migration of the tip, tear, inadvertent puncture, and connection loosening [6,8]. With newer generation catheters, catheter-related complications, such as puncture or migration, are less common [9,10]. However, even though less common, catheter malfunction, especially catheter microfracture, must be kept in the differential when troubleshooting an IDDS [11].

In this case, troubleshooting the IDDS permitted the collection of cerebrospinal fluid (CSF) prior to and after treatment of a patient with acute opioid withdrawal (OW), allowing for the first-in-human testing of the hypothesis of a “memory trace” in the CSF, which can induce a form of long-term potentiation at C-fiber synapses [12]. Upon activation, neurons and glia release inflammatory mediators that are able to alter synaptic strength [12-15]. We hypothesized that there may be differences in levels of inflammatory cytokines in the CSF pre- and post-treatment of OW and that these may correlate with differences in electrophysiological properties of CSF-treated human-induced pluripotent stem cell (hiPSC)-derived nociceptors. The assumption is that these differences may lead to the changes in pain pathways that may contribute to OW symptoms seen in patients who have IDDS malfunction. In understanding these physiological pathways, it is hoped that markers could be identified to understand the pathophysiology and to track treatment.

To date, this is the first reported case to postulate OW that may be due to catheter microfracture not detected using conventional interrogation of the IDDS components. It is also the first-in-human investigation of the effects of CSF pre- and post-treatment of OW to determine possible physiological determinants of OW related to the CSF.

## Case presentation

Participant recruitment

The patient provided verbal and written informed consent. Verbal approval was attained at the time of presentation from the patient to report her case and results in a case report format.

Implanted intrathecal drug delivery system

The products used were Medtronic SynchroMed II Pump (Medtronic, Minneapolis, MN), Medtronic Intrathecal Catheter Spinal Segment Revision Kit, and Medtronic Indura 1PC Intrathecal Catheter.

Cell culture method

The hiPSCs [16] were received from Anatomic Corp (Minneapolis, MN) and stored at -80°C. Glass coverslips were coated with poly-D-lysine and Chrono^TM^ Matrix 3 (Anatomic Corp). Cells were grown in Chrono^TM^ Senso-MM complete growth medium (Anatomic Corp). Cells were plated at 105 cells/cm^2^ and incubated at 37°C, 5% CO_2_, and 95% humidity. Culture media were replaced the day after plating and then every two days thereafter. Cells appeared dorsal root ganglia-like and fired action potentials shooting over +20 mV by day 7 in vitro.

Whole-cell patch-clamp electrophysiology analysis method

HiPSC-derived nociceptors were identified by infrared-differential interference contrast (IR-DIC) microscopy using an Olympus digital camera. Current clamp recordings were performed as previously described [17].

Cytokine analysis method

A total of 200 µL/sample was analyzed using the Human Cytokine Array Kit (R&D Systems, Minneapolis, MN) and imaged using an Odyssey Fc Imaging System (Li-Cor, Lincoln, NE) with an exposure time of 2 minutes for optimal contrast. Fluorescence intensity values measured using ImageJ.

Statistical analysis method

Statistical analysis was performed with GraphPad Prism 8.0 (GraphPad Software Inc., La Jolla, CA). Statistical significance attributed results yielding p-value < 0.05.

Patient presentation

A 46-year-old female post-IDDS placement for failed back surgery syndrome presented for signs and symptoms concerning for OW. The patient’s pain had increased from her baseline of 2/10 to 9/10. She complained of approximately 24 hours of insomnia, anxiety, severe tremors, bilateral lower extremity myalgias and arthralgia, nausea, non-bloody emesis, diarrhea, and sensation of being flushed. She denied any new focal neurological symptoms. Her Clinical Opiate Withdrawal Scale score was 27 (moderately severe) [18]. Vital signs were significant for a blood pressure of 125/104 mm Hg, a pulse of 125 beats per minute, and a respiratory rate of 23 breaths per minute.

She reported only taking hydrochlorothiazide for chronic hypertension at home. The pump was programmed to bolus 60 mcg fentanyl/0.75 mg bupivacaine over two minutes without relief. She received 4 mg intravenous (IV) hydromorphone followed by 50 mcg IV fentanyl in the emergency room. She reported that her symptoms somewhat improved with the IV medications, but she continued to be in visible distress.

**Figure 1:**
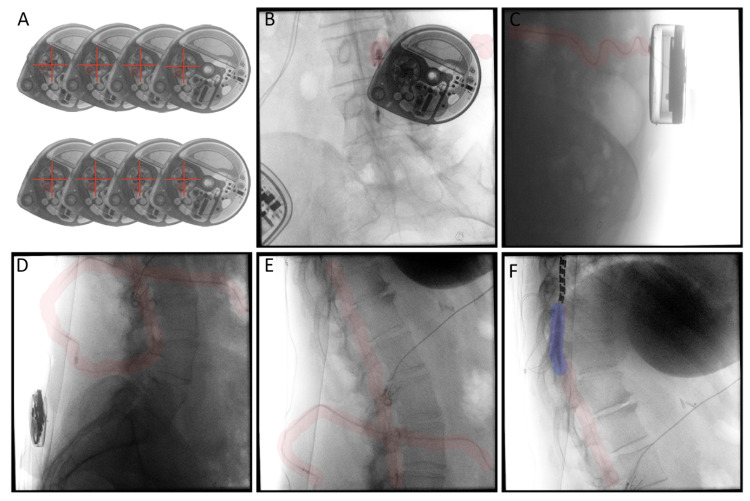
Fluoroscopic Dye Study. (A) Eight time-lapse images of the Medtronic SynchroMed II Pump motor, demonstrating no issues with rotor function; red crossbar and circle have been added to aid with visualization of rotation. (B) Dye study needle in the side port well with no extravasation of contrast around the Medtronic SynchroMed II Pump; red highlight outlines the catheter. (C-F) No extravasation from the catheter; red highlight outline catheter and connection. (F) Catheter tip at the T9 level with a gravity-dependent spread of contrast into the thecal space (purple highlight). Note the presence of a spinal cord stimulator.

The patient was taken to the operating room on the night of presentation for catheter dye study (Figure 1), pump roller study, and reservoir check. “Pre-treatment” CSF sample was attained at this time. No catheter leaks were visualized (Figures 1B-1F), and the rotor study demonstrated correct pump function (Figure 1A) with no error messages from pump interrogation. The catheter tip was at the T9 level, with no evidence of catheter migration (Figure 1F). The contrast layered in a gravity-dependent manner in the thecal space without radiographic evidence of granuloma (Figure 1F). After every other possible explanation was ruled out, it was hypothesized that signs and symptoms may be secondary to catheter microfracture, and the patient was scheduled for catheter replacement.

**Figure 2:**
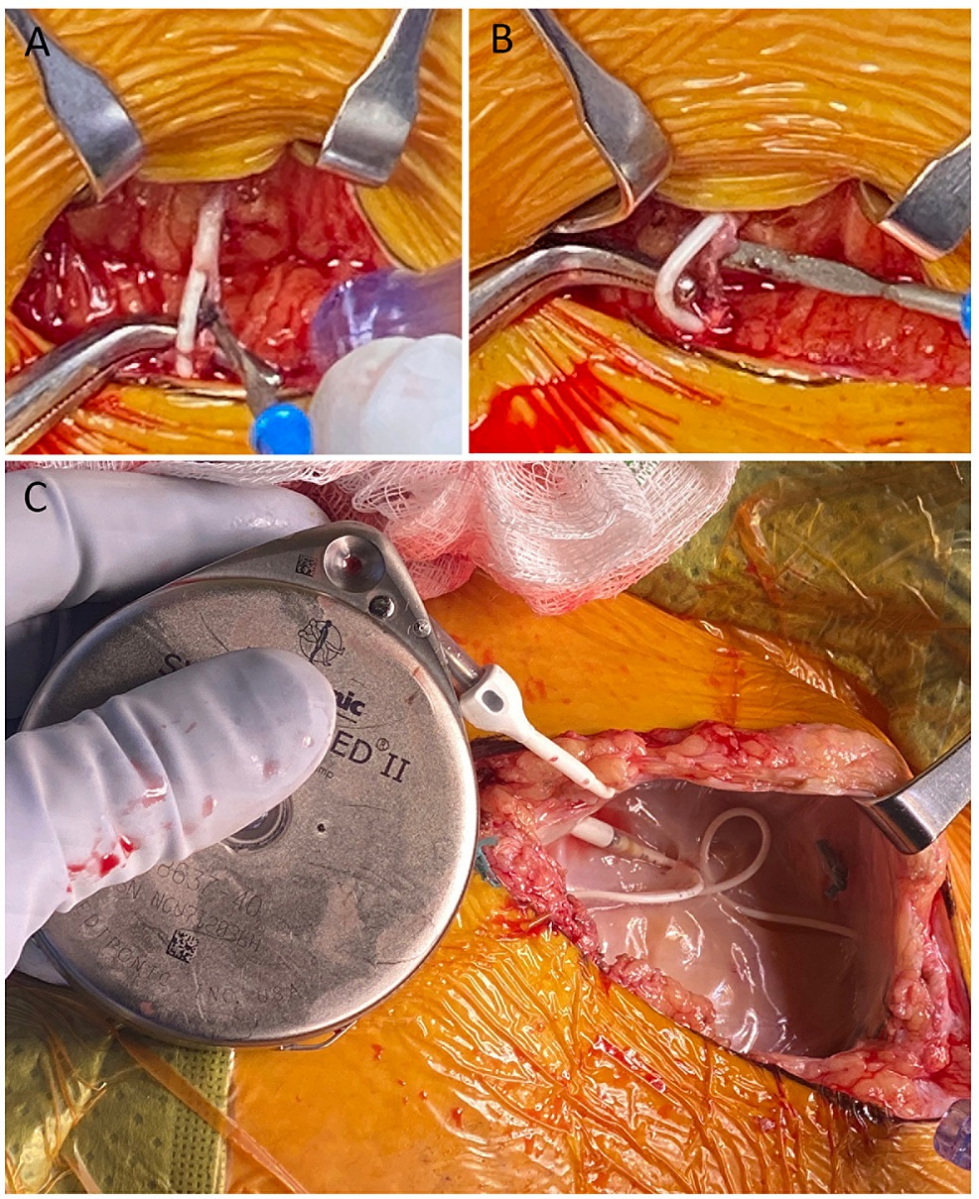
Surgical Images. (A and B) Images of the catheter with biological material encapsulating the catheter; no gross structural defects were observed. (C) Medtronic pump; no gross structural defects were observed.

While waiting to return to the operating room, her scheduled and PRN (pro re nata) medications included IV hydromorphone patient-controlled analgesia 0.5 mg, IV hydromorphone 0.5 mg, clonidine 0.1 mg PO (oral), IV diphenhydramine 12.5 mg, and IV Ativan 1 mg. The following day, OW symptoms had resolved. The patient underwent a pocket revision of ITP and catheter reimplantation (Figure 2). “Post-treatment” CSF sample was attained at this time. Pain was successfully controlled with the IDDS after a new catheter was implanted, and the patient has not required further PO pain medications since the revision.

Whole-cell patch-clamp electrophysiology analyses

**Figure 3:**
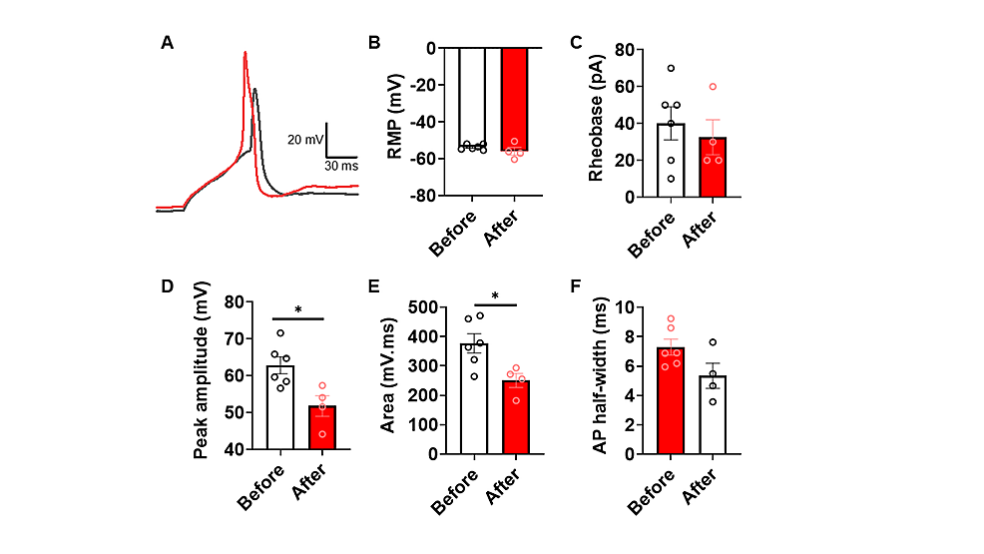
Electrophysiological Analyses of hiPSC-Derived Nociceptors Treated With Patient CSF Before and After Treatment. (A) Representative current-clamp recordings showing AP waveforms from hiPSC-derived nociceptors treated with CSF from the patient before (red) and after (black) treatment. Comparison of (B) RMP, (C) rheobase (current injection required to elicit firing), (D) AP peak amplitude (mV), (E) area under AP waveform (mV.mS), and (F) AP half-width (ms) of APs recorded at rheobase current injection for hiPSC-derived nociceptors treated with CSF from the patient before (red) and after (black) treatment. Statistically significant increases were observed for peak amplitude (p=0.019, Mann-Whitney test) and area (p=0.0381, Mann-Whitney test) for nociceptors treated with CSF from before compared to after treatment. This is indicative of modulation of ion channel activity controlling AP shape that may lead to increased excitability when the patient reported higher VAS pain scores. n=four to six neurons per condition. AP, action potential; CSF, cerebrospinal fluid; hiPSC, human-induced pluripotent stem cell; RMP, resting membrane potential; VAS, visual analog scale

In order to determine if there was a differential effect of pre- and post-treatment CSF on neuronal firing properties, we studied the effect of CSF on hiPSC-derived nociceptor electrophysiological properties (Figure 3). Cells were treated with a 1:10 dilution of CSF to prevent any osmotic effects [19,20]. Limited by CSF quantity, experiments were limited to two batches of cell cultures (n=four to six neurons per condition). Cells treated with pre- and post-treatment CSF showed no change in resting membrane potential, rheobase (current required to elicit firing), or action potential half-width (p>0.05, Mann-Whitney tests), but a noticeable decrease in both action potential peak amplitude (p=0.019, Mann Whitney test) and area under the curve was observed (p=0.0381, Mann-Whitney test).

Cytokine analyses

**Figure 4:**
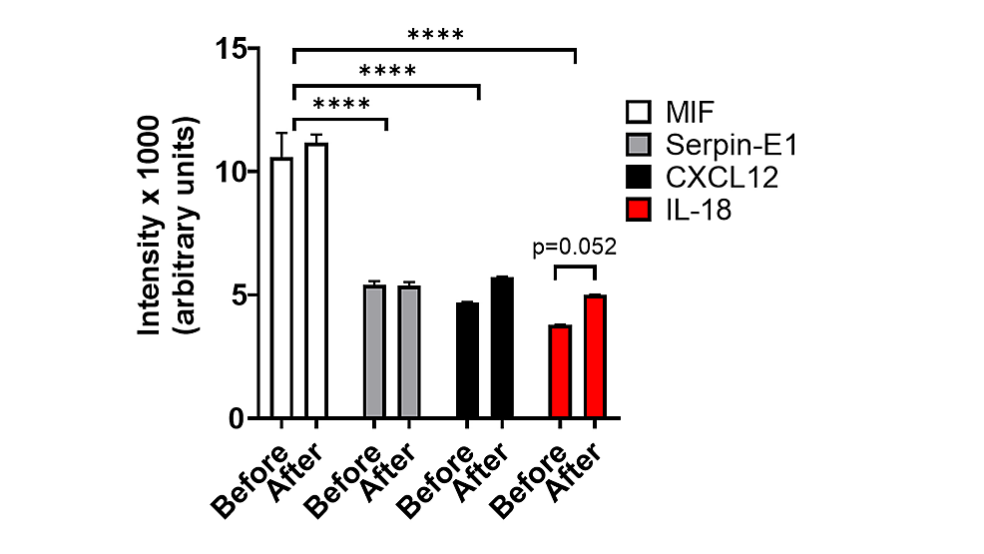
Cytokine Levels From Patient CSF Before and After Treatment. Only levels of MIF, Serpin-E1, CXCL12, and IL-18 were detected. We did not note any significant differences in cytokine levels between patient CSF from before and after treatment, but did note that levels of MIF were significantly higher than all other cytokines. ****p<0.0001, two-way ANOVA with Tukey’s multiple comparisons test. CXCL12, C-X-C motif chemokine ligand 12; IL-18, interleukin-18; MIF, migration inhibitory factor; Serpin-E1, serine protease inhibitor E1

Cytokine analysis was conducted to determine if there was a differential expression of cytokines in pre- and post-treatment CSF. Macrophage migration inhibitory factor (MIF), serine protease inhibitor E1 (serpin-E1), C-X-C motif chemokine ligand 12 (CXCL12), and interleukin-18 (IL-18) were detected by the assay (Figure 4). We did not detect significant differences in any of these cytokines between CSF obtained before and after treatment, although levels of IL-18 were close to being significantly higher in CSF from pre- versus post-treatment (p=0.052, two-way analysis of variance [ANOVA] with Tukey’s multiple comparisons test).

MIF levels were significantly higher than serpin-E1, CXCL12, and IL-18 (p<0.0001, two-way ANOVA with Tukey’s multiple comparisons test). These data suggest that MIF may be significantly upregulated in patients with IDDS to control chronic pain.

## Discussion

In this case study, we present the first case report of a patient undergoing acute OW secondary to assumed catheter microfracture. We suspect microfracture of the catheter, as live fluoroscopic interrogation of the IDDS revealed no malfunction of the pump (as the pump motor was interrogated and observed to turn appropriately), no issues with catheter patency (as CSF was aspirated from the access port), no contrast leakage at the catheter connection site as well as no contrast leakage along the length of the catheter (with live fluoroscopy and digital subtraction imaging), and no subdural extravasation at the catheter tip. Moreover, the contrast pattern at the tip of the catheter ruled out the possibility of occlusion at the tip by granuloma formation. The most convincing evidence excluding the Medtronic SynchroMed II Pump and implicating catheter malfunction was the lasting resolution of symptoms upon catheter replacement. The authors of this case report agree with Dawes et al. that the presence of catheter microfractures may be intermittently patent, leading to unremarkable routine investigations [11]. It may be quite difficult or nearly impossible to demonstrate microfractures in vivo, as issues may be dependent on body position and the malfunction cannot be observed without scanning electron microscopy (SEM) [11].

This case highlights the importance of keeping catheter microfracture in the differential, with unremarkable IDDS interrogation. As previously proposed by Dawes et al. “careful search of the catheter under magnification [with appropriate magnification under scanning electron microscopy] should also be done to look for small fractures when revising a catheter, particularly when the symptomatology is confusing and a microfracture suspected” [11]. The authors propose that SEM should become part of routine investigations of IDDS failure, when other possible explanations are ruled out and microfracture is suspected. It was a shortcoming of our interrogation that we did not request for the manufacturer to conduct further testing or request the manufacturer to supply us with the catheter for in-house investigations using SEM. By presenting this case study, we hope to encourage others not to make the same mistake.

The differential effects of pre- and post-treatment CSF indicate altered ion channel activity controlling AP shape that may lead to increased neuronal firing affecting reported pain in the patient [21]. Macrophage migration initiation factor (MIF) was upregulated in both pre- and post-CSF samples when compared to other cytokines detected. MIF is positively correlated with the severity of bladder pain [22-24], endometriosis [25], and osteoarthritis [26]. However, no clear treatment differences in cytokine levels of pre- and post-CSF samples were observed, which may be due to lack of sensitivity of the assay. Further experiments using more sensitive methods (such as mass spectrometry) may be required to measure cytokine levels in pre- and post-treatment CSF. The electrophysiological changes we see in nociceptors treated with CSF may be due to soluble mediators in patient CSF other than cytokines or cytokines at levels that could not be detected using our cytokine assay.

## Conclusions

In patients presenting with acute OW attribution IDDS malfunction, catheter microfracture must be in the differential, and non-conventional interrogation of the IDDS catheter should be considered. The possible differences in pre-CSF and post-CSF may be more complicated than previously postulated as there were no significant differences in cytokine profiles; however, treatment of in vitro neurons with pre- and post-CSF resulted in differential neuronal excitability, which may account for some of the symptoms of OW.
